# Quantum Dot-Doped Electrospun Polymer Fibers for Explosive Vapor Sensors

**DOI:** 10.1021/acsanm.3c00370

**Published:** 2023-05-19

**Authors:** Dalton Ennis, Dylan Golden, Mackenzie C. Curtin, Alma Cooper, Cynthia Sun, Kathleen Riegner, Caleb C. Johnson, Julia L. Nolletti, Kingsley B. Wallace, Jose A. Chacon, Haven Bethune, Tessy S. Ritchie, Vincent Schnee, Daniel R. DeNeve, Dawn E. Riegner

**Affiliations:** ‡ Department of Chemistry and Life Science, United States Military Academy, West Point, New York 10996 United States; ϕ Fires Center of Excellence, United States Army, Fort Sill, Oklahoma 73503 United States; Δ Department of Chemical Engineering & Materials Science, Stevens Institute of Technology, Hoboken, New Jersey 07030 United States; Ω C5ISR Center Night Vision and Electronic Sensors Directorate, U.S. Army Combat Capabilities Development Command, Aberdeen Proving Ground, Maryland 21005 United States; ¥ U.S. Army, 3rd Infantry Division, Fort Stewart, Georgia 31314 United States; § U.S. Army, Combined Arms Support Command, Fort Lee, Virginia 23801 United States; β Center for Devices and Radiological Health, U.S. Food and Drug Administration, Silver Spring, Maryland 20993 United States

**Keywords:** explosives sensor, explosives detection, nanofibers, polymers, explosives, quantum dots

## Abstract

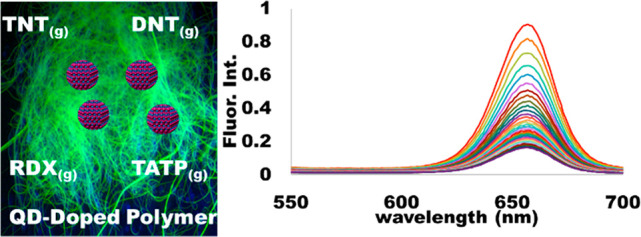

This research seeks to support reconnaissance efforts against homemade explosives (HMEs) and improvised explosive devices (IEDs), which are leading causes of combat casualties in recent conflicts. The successful deployment of a passive sensor to be developed for first responders and military must take expense, training requirements, and physical burden all into consideration. By harnessing the size-dependent luminescence of quantum dots (QDs) being electrospun into polymer fibers, the authors of this work hope to progress toward the development of lightweight, multivariable, inexpensive, easy to use and interpret, field-applicable sensors capable of detecting explosive vapors. The data demonstrate that poly(methyl methacrylate) (PMMA), polystyrene (PS), and polyvinyl chloride (PVC) fibers doped with Fort Orange cadmium selenide (CdSe) QDs, Birch Yellow CdSe QDs, or carbon (C) QDs will quench in the presence of explosive vapors (DNT, TNT, TATP, and RDX). In all cases, the fluorescent signal of the doped fiber continuously quenched upon sustained exposure to the headspace vapors. The simple method for the integration of QDs into the fibers’ structure combined with their straightforward visual response, reusability, and durability all present characteristics desired for a field-operable and multimodal sensor with the ability to detect explosive threats.

## Introduction

The constantly evolving domains of homeland security and international conflict require increasingly sophisticated explosive detection methods. Hidden explosives are commonly used in domestic and foreign terrorism.^1^ Homemade explosives (HMEs) can be easily produced, with common examples being triacetone triperoxide (TATP) and ammonium nitrate/fuel oil (ANFO).^2^ Improvised explosive devices (IEDs) tend to modify old munitions which typically use trinitrotoluene (TNT) and hexahydro-1,3,5-trinitro-1,3,5-triazine (RDX) as the primary explosives.^3^ The development of robust, cost-efficient, and rapidly employable devices capable of detecting a wide range of explosive vapors thus fulfills an important national security and military capability.^4^

Current detection methods tend to focus on locating explosive containers and include ground-penetrating radars, X-rays, and metal detectors. These devices, however, are unable to detect the presence of an explosive compound. Methods for detecting vapors of explosive agents typically include working dogs, gas chromatography, ion mobility spectroscopy, and amplified fluorescence devices such as Fido.^5−8^ Working dogs require specialized, expensive training, and their applicability is limited by their handler’s ability to transport, house, and sustain them in a myriad of potentially austere and unsuitable environmental conditions.^9^ The use of detection instrumentation is expensive and requires extensive training to operate and interpret results. The burden of carrying some of these devices is not always desired in a reconnaissance effort, and it is cost-prohibitive for each individual responder to have their own device.

The goal of this research is to specifically demonstrate a technology that will detect vapors of explosives and could potentially be developed into a wearable sensor on each first responders’ uniform. This sensor would leverage the properties of quantum dots (QDs) electrospun into polymer fibers, where the polymer fibers could be woven into the uniform or worn as a device.

QDs, first described by Ekimov et al. in 1981, are nanoscale semiconductor crystals that demonstrate size-dependent luminescent properties.^10^ Adjusting synthesis parameters allows tuning of the fluorescent properties to a desired region of the electromagnetic spectrum. QDs comprise a crystal structure widely ranging from hundreds to several thousands of atoms and typically span about one to 30 nanometers (nm). Their unique electronic, optical, and chemical properties emerge from the quantum mechanical effects of the confinement of charge carriers.^11^ QDs were selected as a chemical sensor due to previous research demonstrating the detectability of explosive compounds dissolved in solution through fluorescence measurements.^11,12^

Polymer systems have shown great promise as sensors for nitroaromatics, particularly conjugated polymer systems such as polyacetylenes, poly(p-phenylenevinylenes), poly(p-phenylene ethynylenes), etc.^13^ However, many of these systems involve in-depth synthetic procedures that may result in cost- or time-prohibitive approaches that can limit low-cost and rapid production of sensor materials. As a result, in this study, rather than using a polymer on its own as a sensor, the polymers were used as a vehicle to carry the quantum dots.

Previous studies demonstrate the use of porphyrins embedded in electrospun nanofibers as nitroaromatic explosive detectors,^14^ thus introducing a method for portable optical devices as detectors. Schlecht et al. embedded QDs in polystyrene nanofibers to create linear arrays of QDs for optical response without exploring potential applications.^15^ The work of Li et al. presents a method for metal ion detection using encapsulated QDs.^16^ Mahmoudifard et al. demonstrated the development of functionalized cadmium telluride QD-doped poly(vinyl alcohol) nanofibers for use in detecting environmental pollutants such as benzene, toluene, and xylene.^17^ Nitroaromatics as solids, in solution, and as vapors were detected by functionalized Si-QDs luminescence quenching by Nguyen et al.^18^ The work done by Xiong et al. utilized zinc(II)-coordination nanofibers for fluorescence detection of explosive vapors that were generated by heating the analytes to 140 °C.^19^ Additionally, Wu et al. demonstrated synthesis of electrospun polyurethane fibers which were subsequently loaded with 21% by weight zinc sulfide QDs.^20^ While their concept is similar to that presented here, the number of steps Wu et al. required for fiber preparation and QD loading was both intensive and time-consuming as a result of treating the QDs with adducts for explosive selectivity.

This research looks to address gaps in the literature, namely, detection of explosive vapors under ambient conditions utilizing facile fiber preparation, with simultaneous incorporation of QDs without modification. Presented here is a much more streamlined method to create a fluorescent sensor compared to previous work. Finally, there is a need for more data focused on headspace analysis of explosives measured under ambient conditions to work toward real-world applications. This study presents the initial results of explosive sensing by incorporating various QDs into various polymer fibers through electrospinning. Demonstrable proof of the QDs incorporated into the fibers was carried out using microscopy and spectroscopy. The QD-doped fibers, upon exposure to the headspace vapors of four different explosive compounds (TNT, DNT, TATP, and RDX), were evaluated to determine the quenching of the fluorescence signal due to the chemical exposure. Incorporation of the QDs into polymer fibers will potentially provide a means for creating a multichemical passive detector that could be employed by the military, first responders, and homeland security professionals.

## Experimental Section

### Fiber Solution Preparation

The specific polymers used in this study were chosen based on their lack of fluorescence in the visible region of the electromagnetic spectrum where the QDs fluoresce. All electrospinning solutions were prepared as a total mass of 10 g. The PVC solution was prepared in tetrahydrofuran (THF, ∼6 mL, Sigma-Aldrich), dimethylformamide (DMF, ∼4 mL, Sigma-Aldrich), and 9% by weight (0.916 g) of high molecular weight (HMW) PVC powder (Sigma-Aldrich). The PMMA solution consisted of tetrahydrofuran (THF, 4.49 mL), chloroform (2.69 mL, Sigma-Aldrich), and 20% by weight of 120 K MW PMMA powder (2.00 g, Sigma-Aldrich). The PS solution was made by combining HMW (260 K) PS powder (Sigma-Aldrich) at 14% by weight with THF (∼4 mL) and DMF (∼6 mL). Then, 0.03% by weight CdSe (Birch Yellow), 0.015% CdSe (Fort Orange), and 0.01% C QDs were added to each of these polymer solutions. The CdSe QDs were purchased from Evident Technologies in a solution of toluene which was subsequently removed using a rotary evaporator. The C QDs were purchased from Sigma-Aldrich. The polymer and QD solutions were stirred overnight at 45 °C before electrospinning.

**Figure 1:**
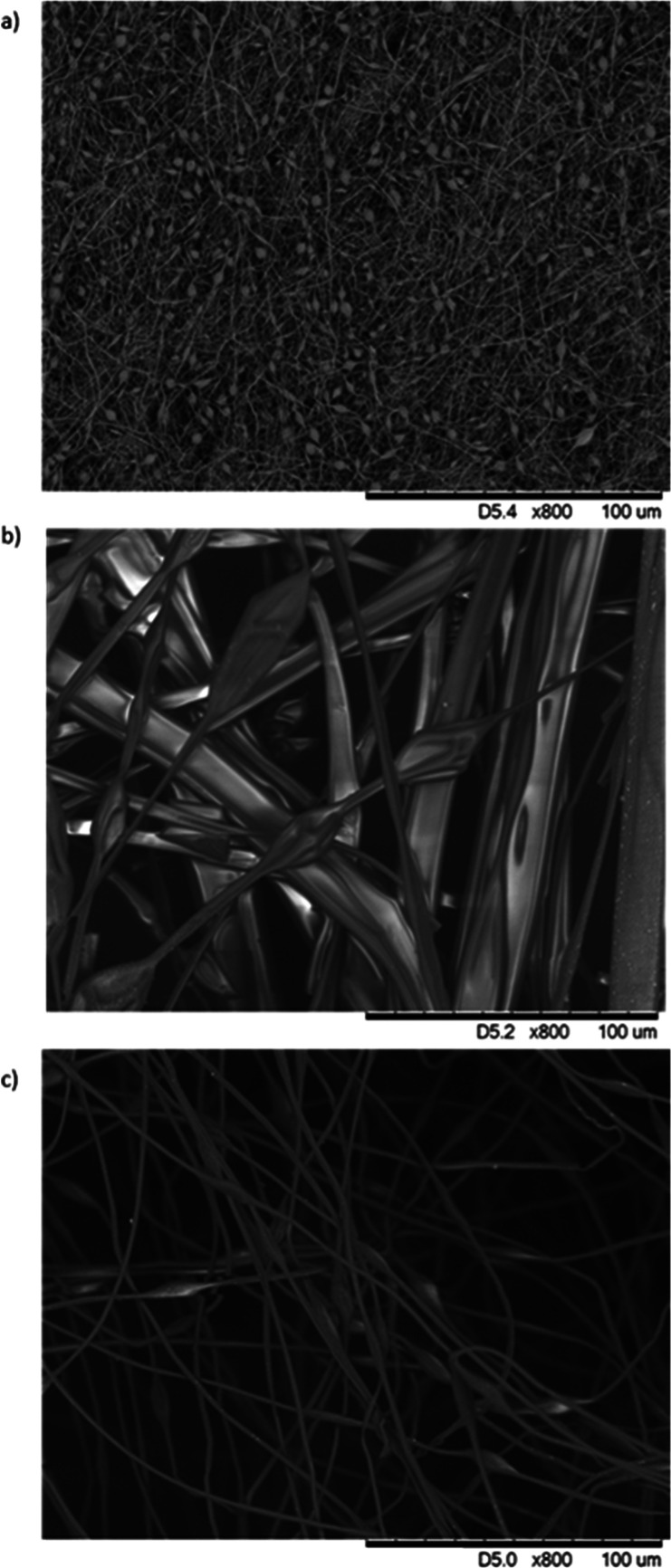
SEM images of a) PVC, b) PMMA, and c) PS with Fort Orange CdSe QDs at 800× magnification.

Polymers with and without QDs were electrospun under the parameters described in Table 1, using the electrospinning setup depicted in Figure S1. After electrospinning, each sample was analyzed using Confocal microscopy and stereomicroscopy. All fibers were viewed under 200× optical magnification to qualitatively assess the uniformity and reproducibility among trials. Figure 1 contains SEM images of each type of fiber that was used after optimizing the electrospinning parameters. Although polymer solution viscosity is a major determining factor in achieving optimized spinning parameters, work has not been done to determine how changes in viscosity can result in different responses to the explosives such as improved efficiency, kinetics, percent quenching, etc.

**Table 1:** Electrospinning Parameters

Polymer	Flow rate (mL/min)	Distance between syringe tip and collection plate (cm)	Potential (kV)
PVC	0.028	17	16
PMMA	0.028	20	16
PS	0.028	15	12

### Explosives

Dinitrotoluene (DNT), TNT, and RDX were all purchased from Sigma-Aldrich. TNT and RDX were purchased dissolved in acetonitrile while DNT was purchased in solid form. TATP was synthesized under strict safety controls to include restricted yield of less than 1 mg, low temperature conditions, and storing in acetonitrile solvent to mitigate risk.^21^

### Instrumental Setup

Explosive solutions were transferred to a piece of cotton in the bottom of a clean cuvette and exposed to a stream of air to ensure evaporation of all solvent. This resulted in a solid mass of explosive ranging from 0.10 mg to 0.50 mg. Solid DNT was weighed and carefully placed in the cuvette. A 0.15 mg to 9.0 mg sample of test fiber was then suspended in the cuvette (Figure S2) at a position that would maximize the overlap of incident radiation with the test fiber without saturating the detector. A cap was placed on the cuvette without further attempts to create a perfect seal. This was done to simulate a realistic dynamic equilibrium with the buildup of vapor pressure, as explosives and their precursors are often found within the confines of buildings, vehicles, or compounds. The cuvette conditions replicate what may be encountered in a realistic scenario. All fluorescence analyses were carried out in a Horiba Jobin Yvon FluoroMax 4 spectrofluorometer at atmospheric pressure with temperatures between 20 and 25 °C and humidity between 25% and 50%.

The doped fibers were excited at the maximum wavelength of absorption for each type of QD (Table 2). Data were collected hourly with a 24 h total exposure time. Upon completion of the experiment, the used explosives were redissolved in acetonitrile and burned in a controlled environment for disposal. The fibers with QDs were deposited in a heavy metal waste container for disposal.

**Table 2:** Fluorimeter Conditions

QD	Excitation wavelength (nm)	Observed emission range (nm)
CdSe (Birch Yellow)	350	500–700
CdSe (Fort Orange)	370	500–700
Carbon	510	500–700^a^

a A portion of this observed emission range is higher energy than the excitation wavelength; there is no expectation of emission below 510 nm.

## Results and Discussion

**Figure 2:**
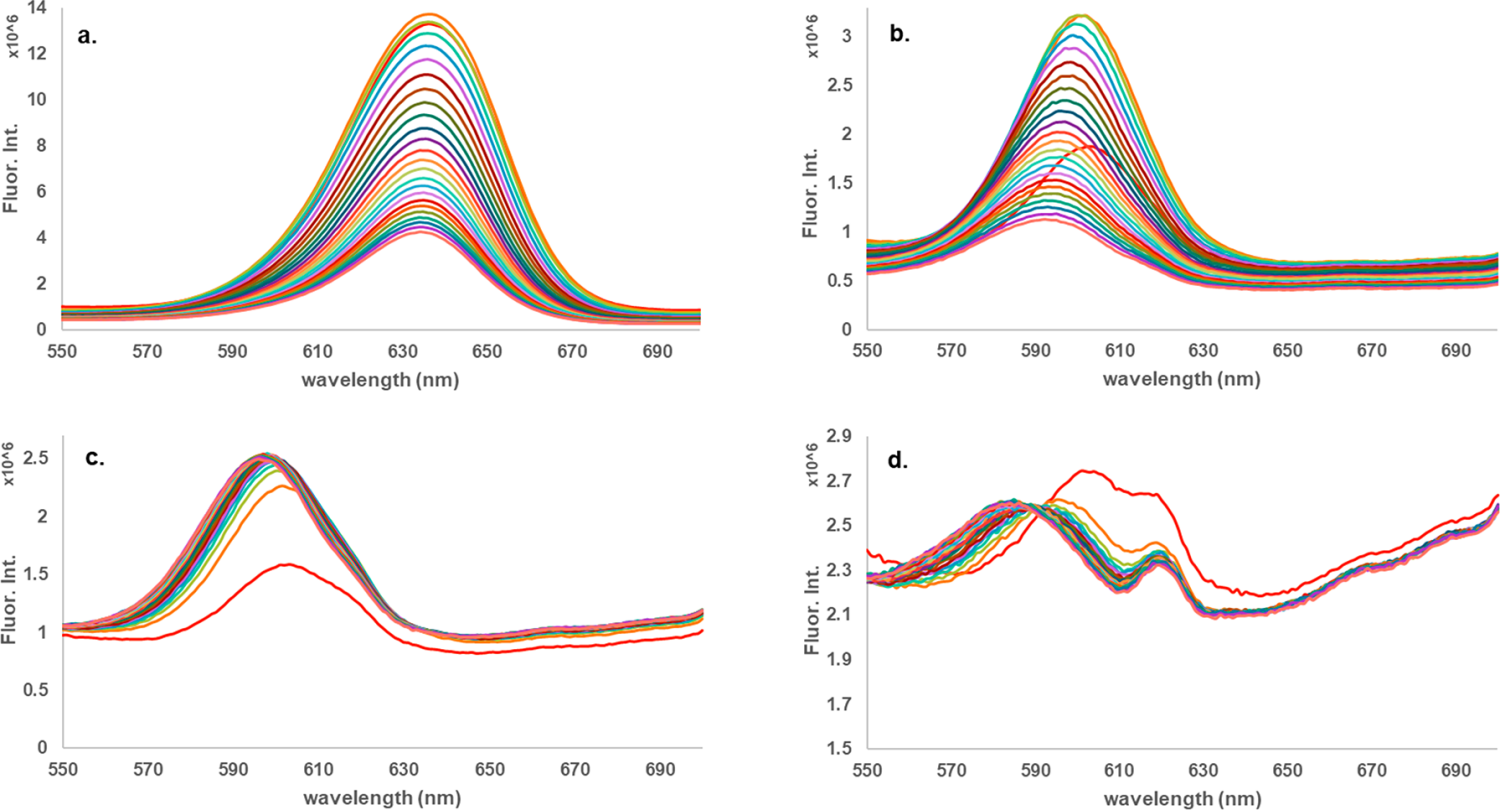
Fluorescence signals of 24-h exposures of PVC doped with Birch Yellow CdSe QDs to various explosives: a) DNT, b) TNT, c) RDX, and d) TATP (See SI for specific color designations for each scan).

**Figure 3:**
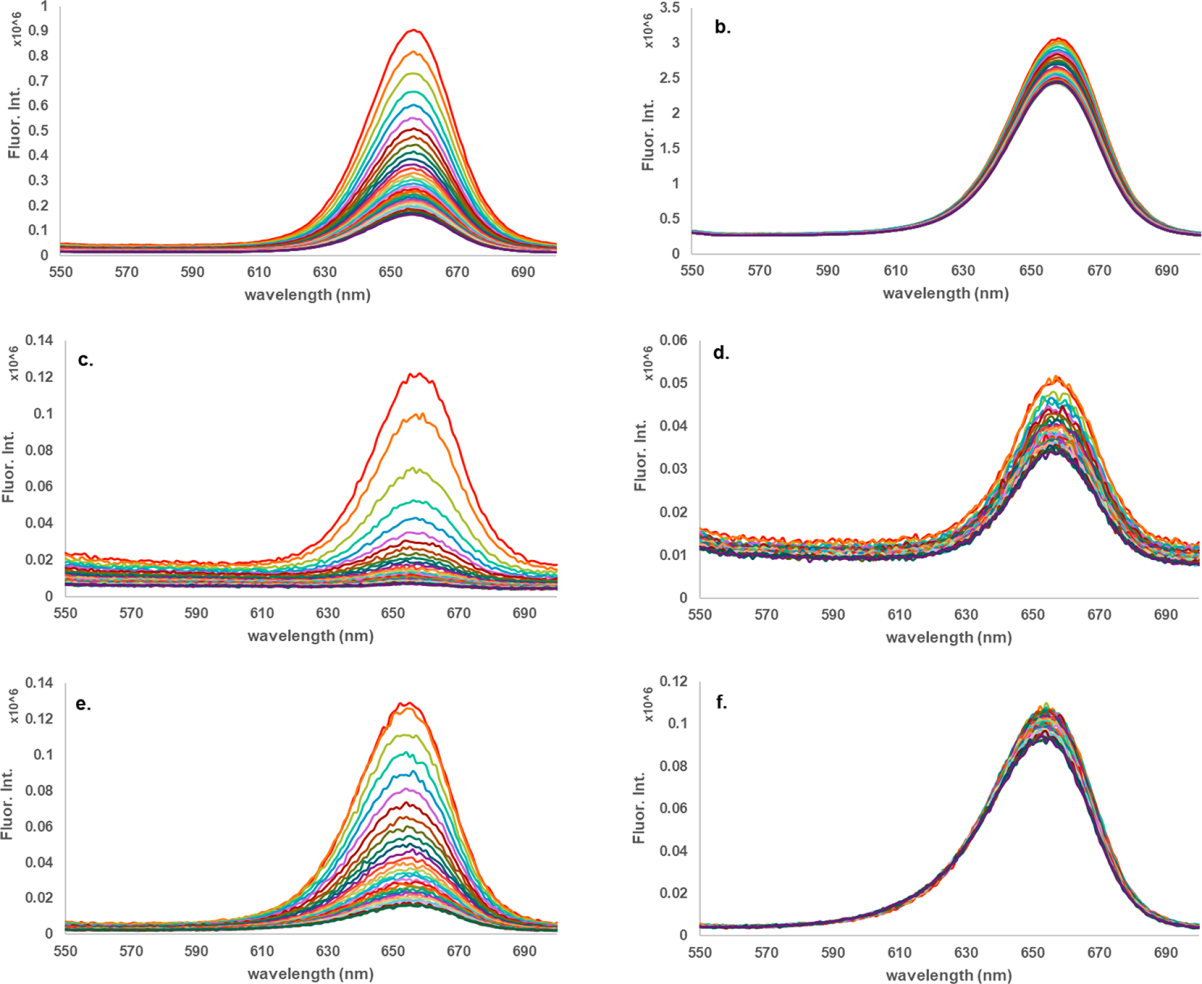
Fluorescence signals of 24-h exposures of Fort Orange QDs in a) PVC with DNT, b) PVC with TNT, c) PS with DNT, d) PS with TNT, e) PMMA with DNT, and f) PMMA with TNT.

Fluorescence emission for fibers with QDs is quenched when exposed to the headspace above the explosives as seen in Figures 2 and 3 (as well as multiple examples in the Supporting Information). There is no fluorescence in the region of interest from the control polymers with no QDs (see Figure S19). Additionally, there is no observed quenching for control fibers with QDs and no explosives (see Figure S20). As seen in Figure 2, PVC doped with Birch Yellow CdSe QDs demonstrates a different emission wavelength depending on which explosive vapors are being detected. This PVC–QD combination demonstrates a signature response to the explosives tested, whereas other combinations (i.e., PVC with Fort Orange CdSe and C QDs) do not show a shift in emission wavelength with explosives tested. This multivariable response to the explosive vapors indicates promise for creating an array of polymers with QDs for the detection of multiple chemicals in a more complex and realistic field environment.

Additionally, the PVC–Birch Yellow combination shows an initial increase in fluorescence between the first two scans (Figure 2a–c, bright red line to orange line) before the fluorescence ultimately begins to quench. Previous studies have demonstrated that the emission intensity of QDs can increase with longer exposure time to a UV excitation source through the phenomenon of irradiation-induced oxygen absorption blocking nonradiative recombination at surface states.^22^ Others have avoided this effect by irradiating their fiber–QD complexes with UV light for hours prior to fluorescence measurements.^20^ We have observed that this occurs only in certain polymer–QD combinations. As seen in Figure 3 when PVC, PMMA, and PS are combined with Fort Orange CdSe, there is no observed increase in fluorescence intensity over time. This can also be seen in Figure S3 for carbon QDs in PVC.

Percent quenching was calculated using the final fluorescence intensity compared to the maximum (1–I_24 h_/I_max_) × 100%. A larger percent quenching indicates a more easily detectable change in fluorescence. The magnitude of percent quenching for each polymer and QD combination per explosive are shown in Table 3. While the exact mechanism of quenching is complex, it is hypothesized that the quenching response is multivariate and includes factors such as polymer structure and permeability, QD composition, vapor pressure of the explosive and its chemical composition, and the previously mentioned UV-induced excitation of the QD–fiber composite.

**Table 3:**
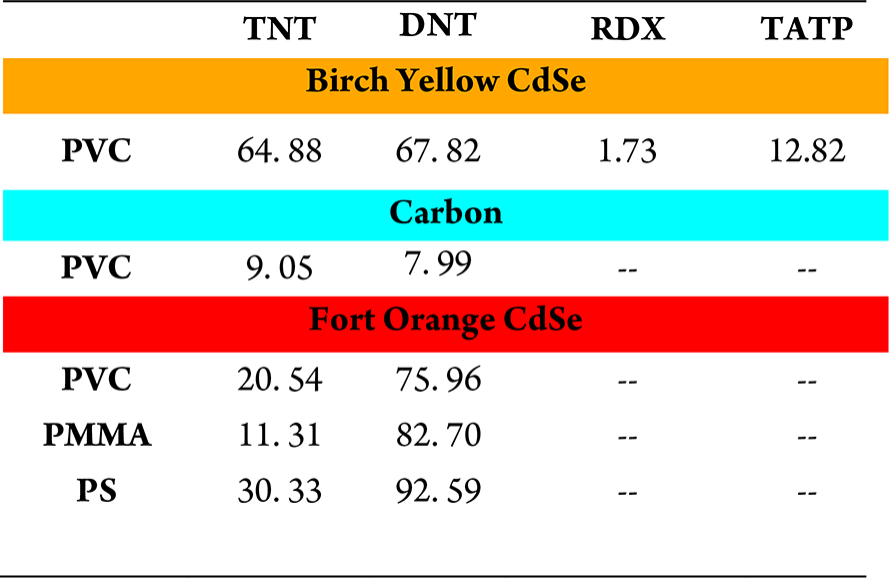
Percent Quenching for All Polymer–QD Combinations

Each polymer’s chemical environment may change the QDs’ quantum mechanical response.^23^ It is hypothesized that the quantum mechanical aspects of the polymer with the QDs are likely to contribute to the quenching mechanism, as indicated by the difference in percent quenching in Table 3 for the three polymers with Fort Orange CdSe QDs and DNT. PS exhibited nearly complete quenching, whereas PMMA and PVC demonstrated a lower magnitude of quenching. This mechanism alone cannot be proven to account for the difference in quenching because the morphology and permeability of the polymer fiber also play a role. The SEM images shown in Figure 1 demonstrate the differences in spun polymer morphology. Polymer structure, based on its spun morphology, impacts the accessibility of the QDs to the explosive vapors.^19^ Additionally, Mahmoudfard et al. reported increased sensitivity with narrowing of the fiber diameter.^17^ More work needs to be done to determine the degree to which structure and morphology affect the magnitude of quenching. This can be accomplished by controlling electrospinning variables to create different morphologies as well as using polymers with comparable functional groups to determine the effect. The size and nature of the QDs in the sensor will deterministically contribute to the degree of quenching. The relative sizes of the QDs are confirmed by their relative emission wavelengths.^10,11,24^ In this study, it appears that QD size is correlated with the percent quenching observed when PVC was combined with each QD and challenged with DNT (Table 3). Blue-green C QDs had the lowest percent quenching, followed by Birch Yellow CdSe QDs, while Fort Orange QDs had the largest percent quenching. This may be true for TNT exposure as well if the experiment controlled for vapor pressure. The difference in quenching between the C QDs and the Birch Yellow CdSe QDs was larger than may be explained by size alone. Further studies examining QDs with the same emission wavelengths but different composition (i.e., blue-green C QDs and blue-green CdSe QDs) could elucidate differences in quenching resulting from the type of QDs.

Previous studies have noted the role of explosive composition on quenching.^11,12,19^ Because previous studies were typically done in solution, the number of nitro groups were directly related to percent quenching.^12^ While this effect still matters in this study, the vapor pressure of the explosives appeared to play a larger role in quenching. This was primarily noted by the fact that DNT yielded a higher percent quenching, despite having fewer nitro groups. When comparing TNT and RDX, which have the same number of nitro groups, the higher vapor pressure of TNT results in higher percent quenching.

In addition to percent quenching, the rate of quenching was observed and can be used to distinguish the various combinations of polymer, QD, and explosive. Previous studies evaluated kinetics by attempting to control vapor pressure with temperature.^19,20^ UV-induced excitation is also a confounding variable that other research groups tried to control by pre-exposing their sensor for up to half a day with UV radiation prior to testing.^19^ While effective, these experimental conditions do not replicate field conditions.

### Influence of Polymers

**Figure 4:**
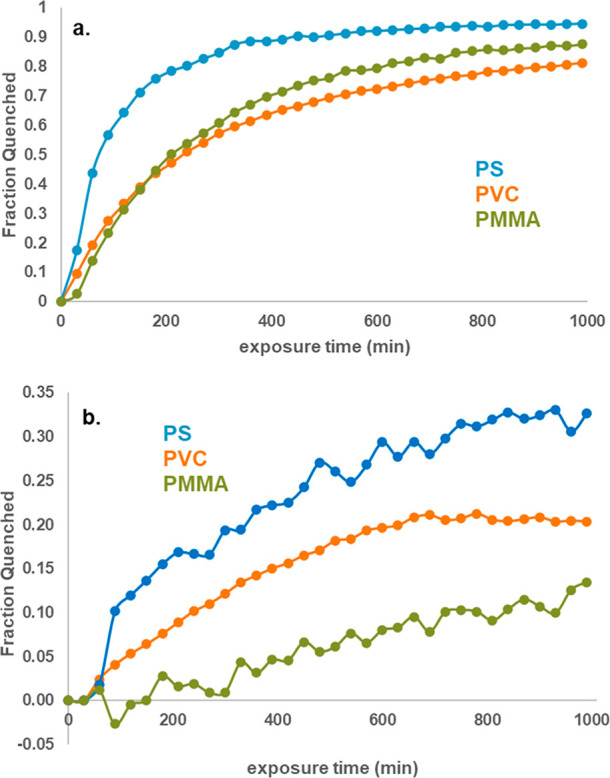
Polymers with Fort Orange CdSe QDs exposed to a) DNT headspace and b) TNT headspace.

As mentioned previously, permeability plays a factor in achieving the maximum percent quenching. Additionally, it plays a role in the kinetics. As shown in Figure 4, all three polymers (PS, PVC, and PMMA) with Fort Orange CdSe QDs were tested against DNT and TNT. Percent quenching was observed at slightly shorter intervals of time to study the kinetics. For both explosives, PS exhibited the fastest response in addition to the previously noted highest percent quenching. It would appear that the polymer structure/morphology plays an important role in the overall response and kinetics. As mentioned earlier, the morphology is determined by the spinning parameters and solution viscosity which are all specific for each polymer solution. As a result, PVC and PMMA parameters were not systematically varied to try to achieve the same spun morphology as PS. Additionally, because the kinetics are complicated, we cannot conclusively state without further study the degree to which vapor pressure and UV-induced excitation influence these results. Controlling for these variables would help to elucidate this information at the expense of the original intent of demonstrating a proof of concept under realistic conditions.

### Influence of QDs

**Figure 5:**
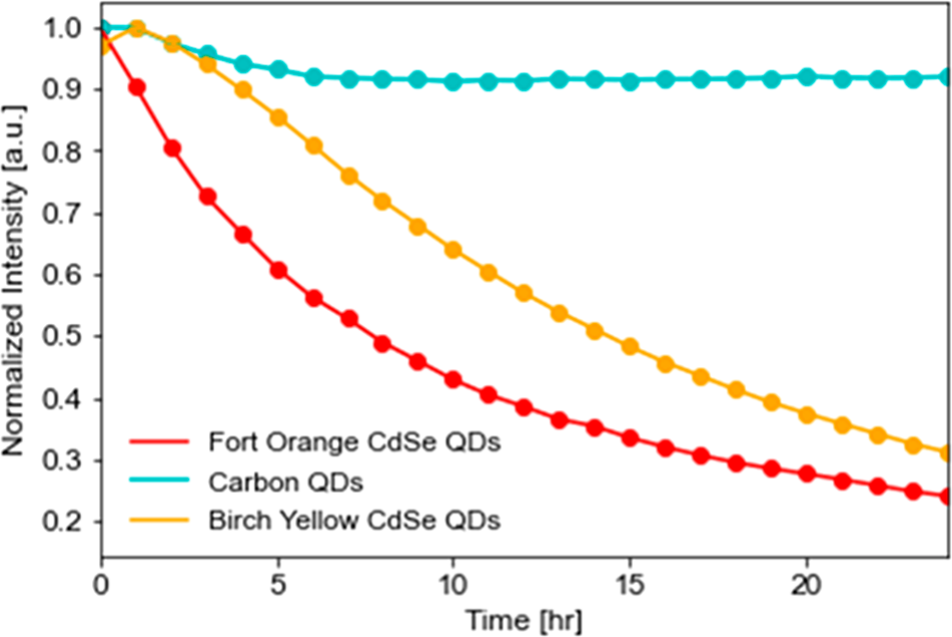
Rates of quenching for each QD in PVC exposed to DNT head space.

In Figure 5, we note the qualitative difference in kinetics between different QDs with the same polymer and exposed to the same explosive. The rate of quenching for C QDs approached final quenching intensity faster during the first few hours as compared to the Birch Yellow and Fort Orange CdSe QDs.

In contrast to the reported percent quenching, the C QDs are almost 1 order of magnitude less responsive than Fort Orange CdSe QDs to DNT. Depending on the time-sensitivity of the detection, the kinetics are an important factor to consider in addition to overall percent quenching.

## Conclusions

**Figure 6:**
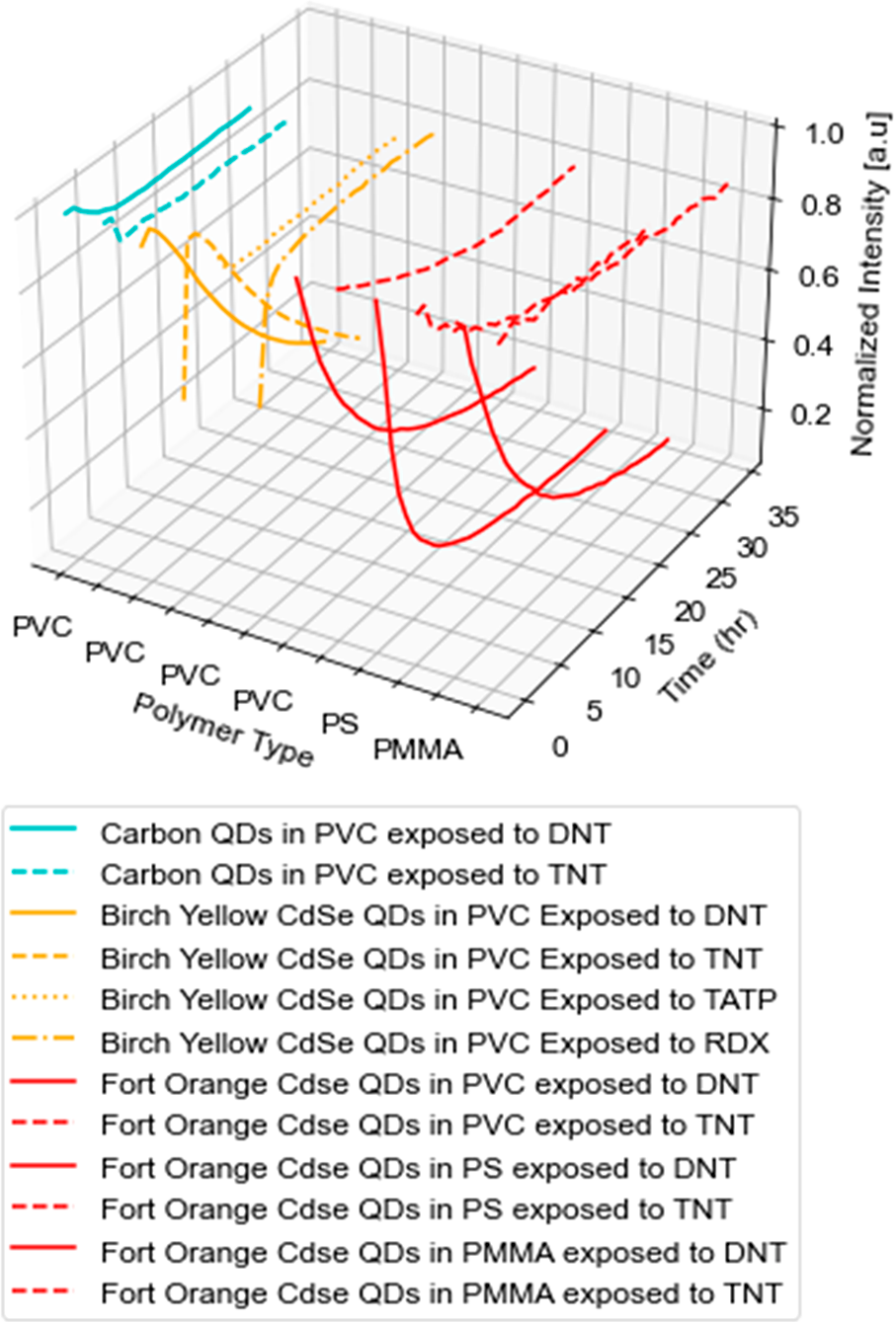
Three-dimensional plot illustrating the three variables.

By incorporating different QDs into PVC, PS, and PMMA, the headspace vapors of TNT, DNT, TATP, and RDX were successfully detected through fluorescence quenching. While quenching was observed for all combinations of polymer and QDs as shown in Figure 6 and Table 3, the varying magnitudes and rates of quenching unique to each system indicate the need for further study with controls in place to isolate the influence of the explosives’ vapor pressures and UV-induced excitation of the polymer–QD system.

While this application shows promise for realistic environmental conditions, optimization of this proof-of-concept in a controlled laboratory setting will require a more rigorous discernment of the multivariate quenching mechanism. Scaling the composition of the sensor, the amount of explosives, controlling environmental conditions (temperature, humidity, and presence of other chemical vapors), and the amount of headspace will increase understanding of the QDs’ capability as a mode of sensing explosives in the field.

Additionally, fiber characterization is also a priority of future work. Although all fibers were spun under optimal parameters (see the experimental section), additional analysis of the structure and morphology of the fibers can facilitate consistent and reliable results. The structure and morphology of the fibers must be considered in relation to ease of QD integration, safety, and the shelf life of the QDs.

To create an ideal sensor, the amount of QDs used must be rigorously optimized. A smaller amount of QDs will quench faster because less explosive vapor is needed to affect a percentage change in quenching. While a larger amount of QDs will have a greater emission intensity, more explosive vapors must be present to achieve a comparable percent quenching. The choice of polymer and QD should achieve an appropriate balance between the kinetics with an accurate measure of percent quenching.

Understanding the extent of the QDs’ ability to sense more functional groups and a variety of other chemicals could facilitate building a catalog of QD detection, making it possible to account for false positives, the presence of benign functional groups, and nonbenign chemicals of interest such as nerve agents or toxic industrial vapors in the environment. With a catalog outlining the quenching profile, it would then be possible to create a multiplexing sensor array which could then provide dynamic confidence intervals as the system continues to evolve. This application could be expanded to enhance situational awareness of chemically complex environments in realistic ambient conditions and potentially scale well to incorporate into a wearable device or as a sensor on a drone platform.

## Supporting Information Available

Experimental design and fluorescence spectra (PDF)
