# Supplementary material for: Quantum Dot-Doped Electrospun Polymer Fibers for Explosive Vapor Sensors
Source: ACS Appl Nano Mater. 2023 May 19;6(11):9315–21. doi: 10.1021/acsanm.3c00370 (PMC10262150; doi:10.1021/acsanm.3c00370)

Supporting Information for

Quantum Dot-Doped Electrospun Polymer Fibers for Explosive Vapor Sensors

Dalton Ennis[‡], Dylan Golden[‡], Mackenzie C. Curtin[§], Alma Cooper[‡], Cynthia Sun[‡], Kathleen Riegner[Δ], Caleb C. Johnson[‡], Julia L. Nolletti[‡], Kingsley B. Wallace[‡], Jose A. Chacon[‡], Haven Bethune[Φ], Tessy S. Ritchie[β], Vincent Schnee[Ω], Daniel R. DeNeve[¥], Dawn E. Riegner[‡]*

[‡]United States Military Academy, Department of Chemistry and Life Science, West Point, NY 10996 USA
[Φ]United States Army, Fires Center of Excellence, Fort Sill, OK 73503 USA
[Δ]Stevens Institute of Technology, Department of Chemical Engineering & Materials Science, Hoboken, NJ 07030 USA
[Ω]U.S. Army Combat Capabilities Development Command, C5ISR Center Night Vision and Electronic Sensors Directorate, Aberdeen Proving Ground, MD 21005 USA
[¥]United States Army, 3rd Infantry Division, Fort Stewart, GA 31314 USA
[§]United States Army, Combined Arms Support Command, Fort Lee, VA 23801 USA
[β]Center for Devices and Radiological Health, U.S. Food and Drug Administration, Silver Spring, MD 20993 USA

*dawn.riegner@westpoint.edu

This PDF includes:
Figures S1 – S20

Figure S1: Electrospinning apparatus

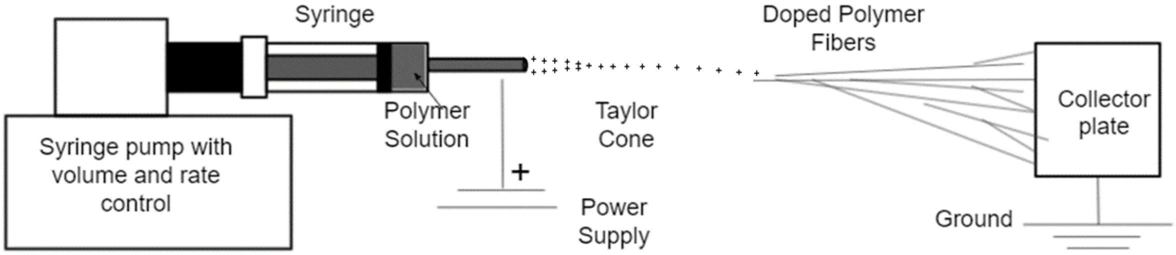

Figure S2: Cuvette with suspension of QD-doped fiber placed above DNT

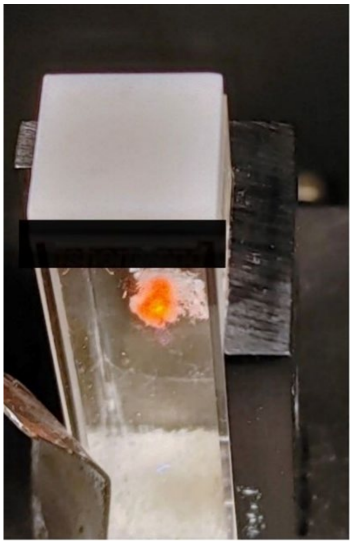

Figure S3: The fluorescence signals of 24-hour exposures of PVC doped with C QDs to various explosives: **a)** DNT **b)** TNT

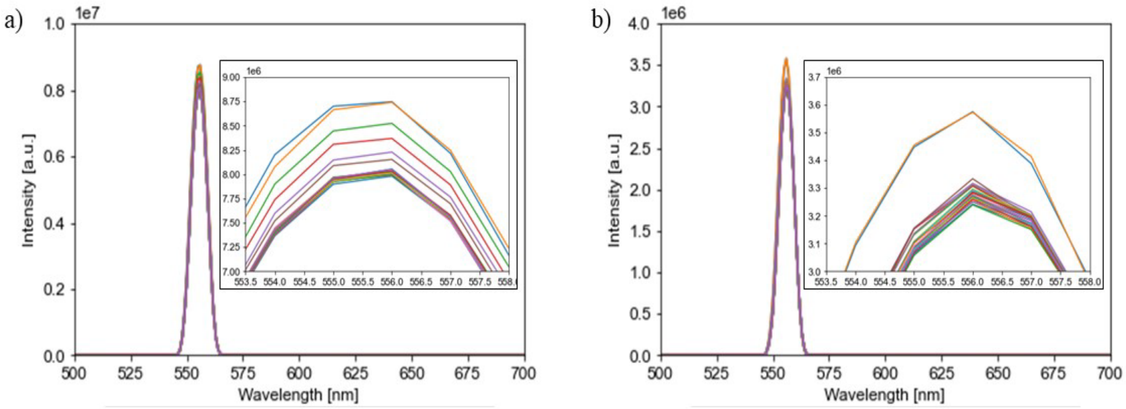

Figure S4: Birch Yellow CdSe in PVC exposed to DNT. 67.82% quenching. a) fluorescence signals every hour b) 0-hour and 24-hour fluorescence signal c) kinetics of fluorescence over time

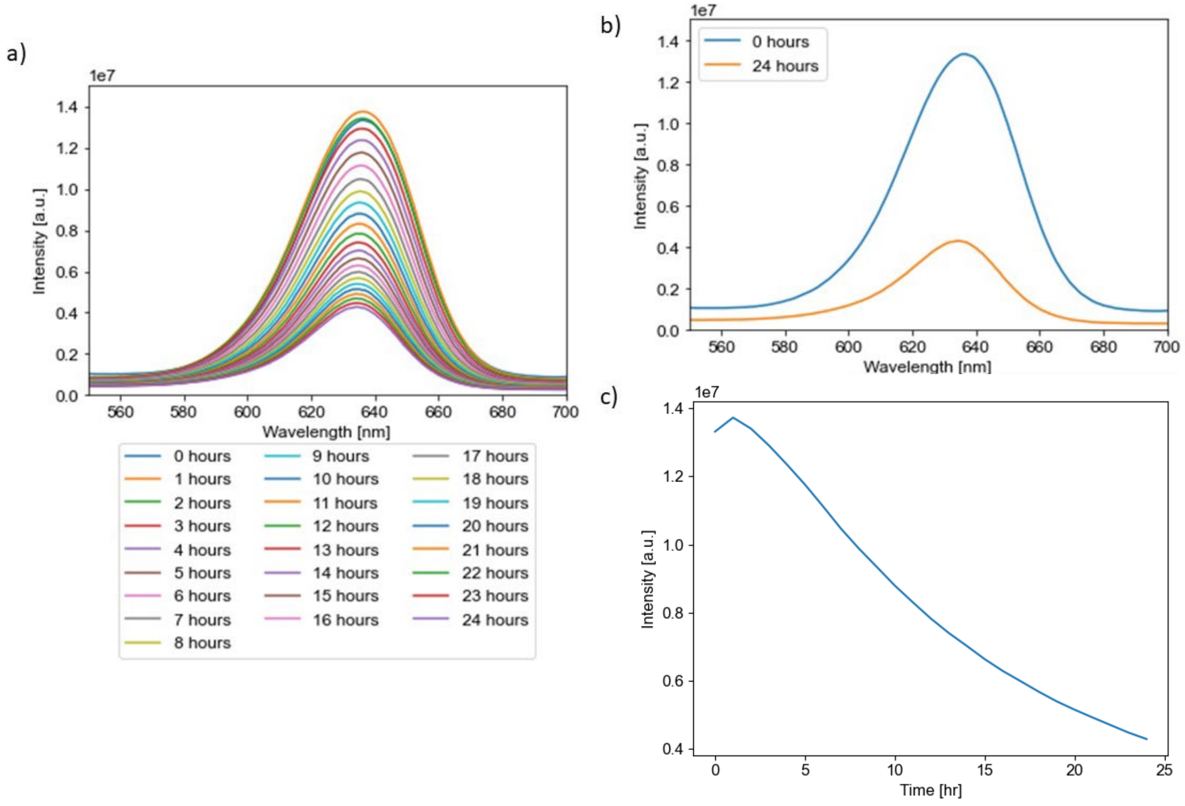

Figure S5: Birch Yellow CdSe in PVC exposed to RDX. 1.73% quenching. a) fluorescence signals every hour b) 0-hour and 24-hour fluorescence signal c) kinetics of fluorescence over time

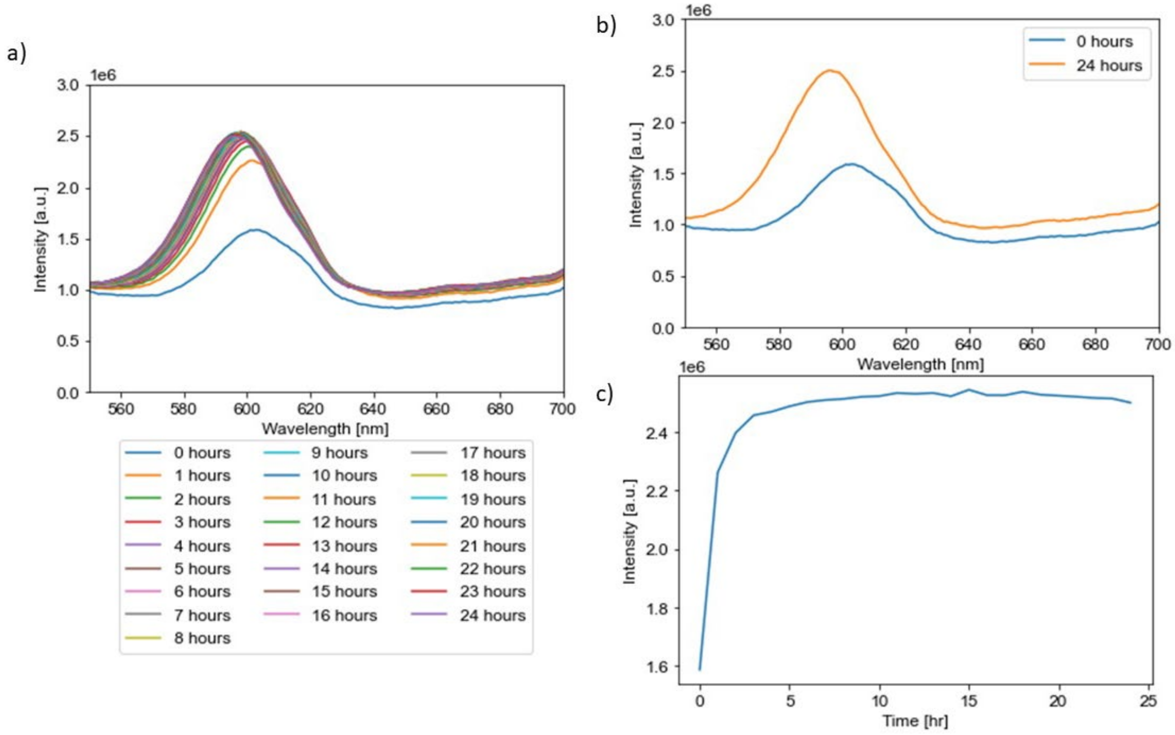

Figure S6: Birch Yellow CdSe in PVC exposed to TATP. 12.82% quenching. a) fluorescence signals every hour b) 0-hour and 24-hour fluorescence signal c) kinetics of fluorescence over time

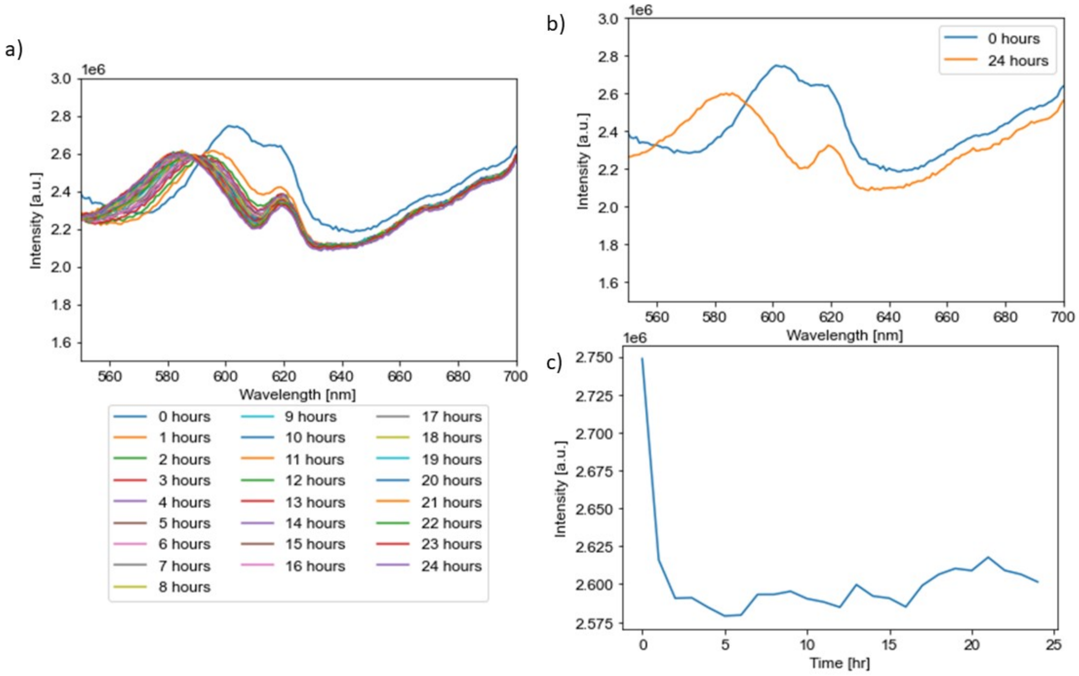

Figure S7: Birch Yellow CdSe in PVC exposed to TNT. 64.88% quenching. a) fluorescence signals every hour b) 0-hour and 24-hour fluorescence signal c) kinetics of fluorescence over time

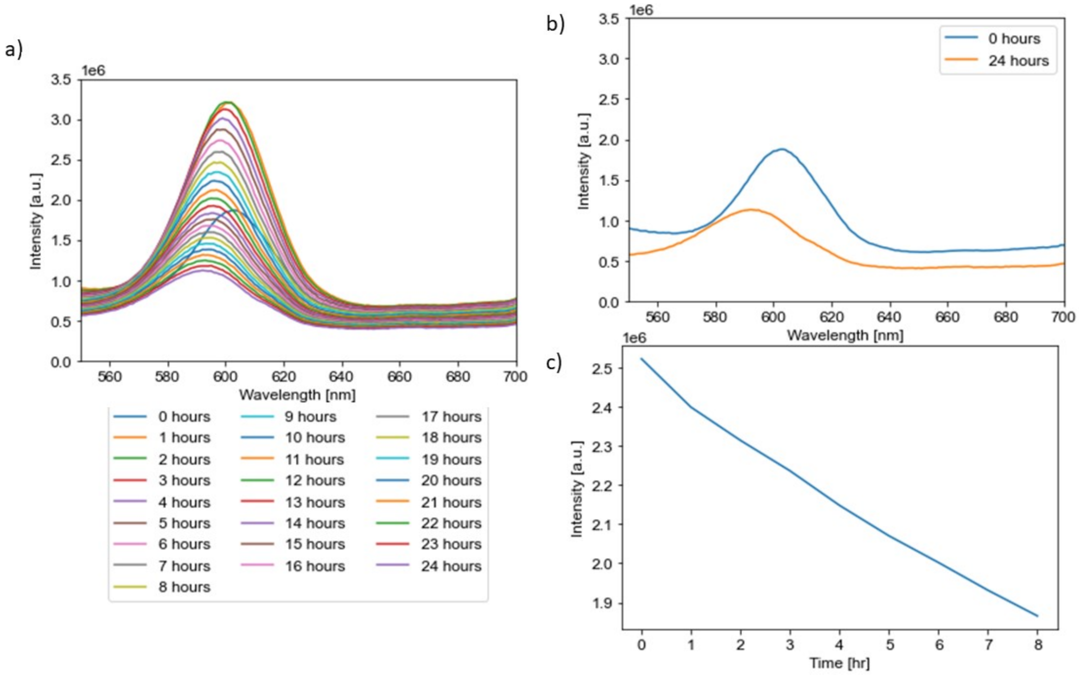

Figure S8: Carbon QDs in PVC exposed to DNT. 7.99% quenching. a) fluorescence signals every hour b) 0-hour and 24-hour fluorescence signal c) kinetics of fluorescence over time

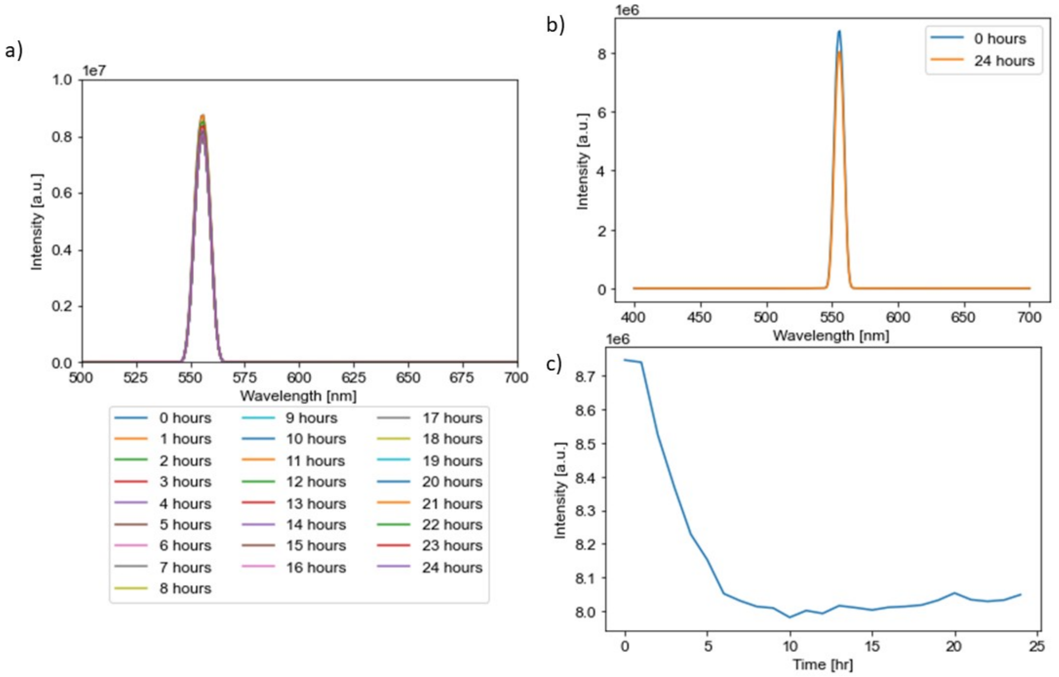

Figure S9: Carbon QDs in PVC exposed to TNT. 9.05% quenching. a) fluorescence signals every hour b) 0-hour and 24-hour fluorescence signal c) kinetics of fluorescence over time

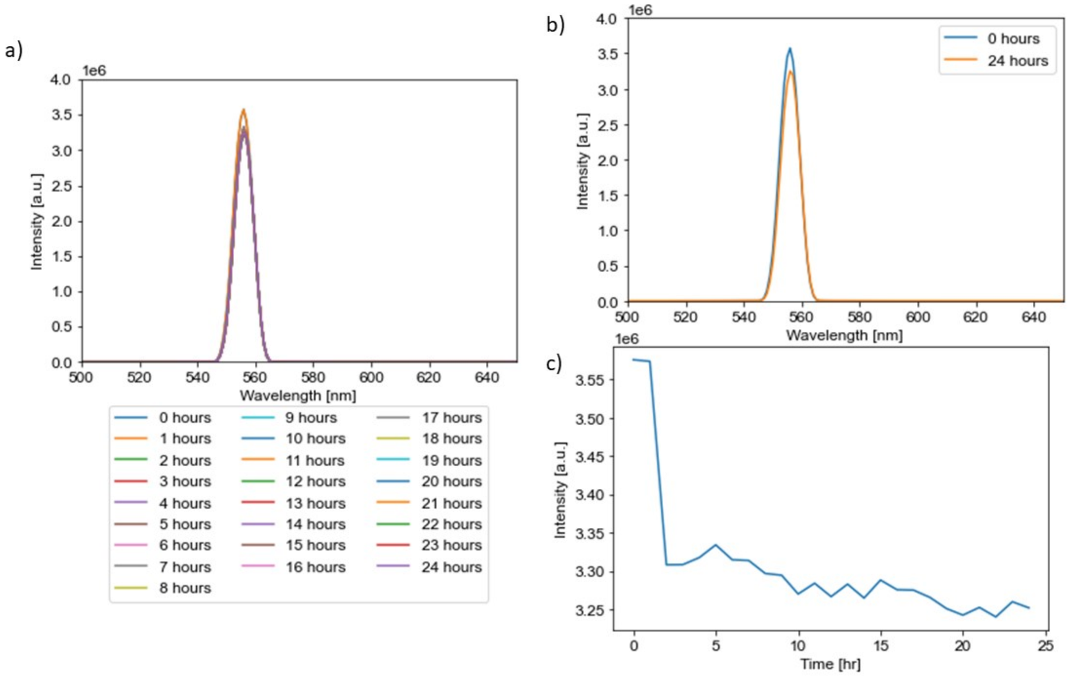

Figure S10: Fort Orange CdSe in PMMA exposed to DNT. 82.70% quenching. a) fluorescence signals every hour b) 0-hour and 24-hour fluorescence signal c) kinetics of fluorescence over time

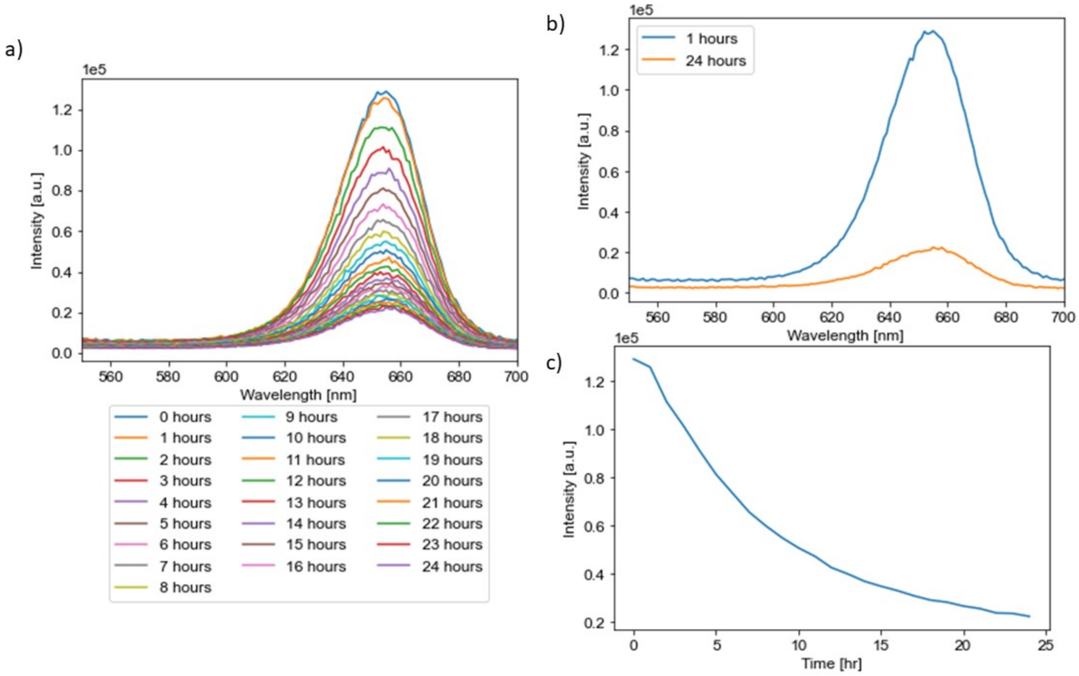

Figure S11: Fort Orange CdSe in PMMA exposed to TNT. 11.31% quenching. a) fluorescence signals every hour b) 0 hour and 24 hour fluorescence signal c) kinetics of fluorescence over time

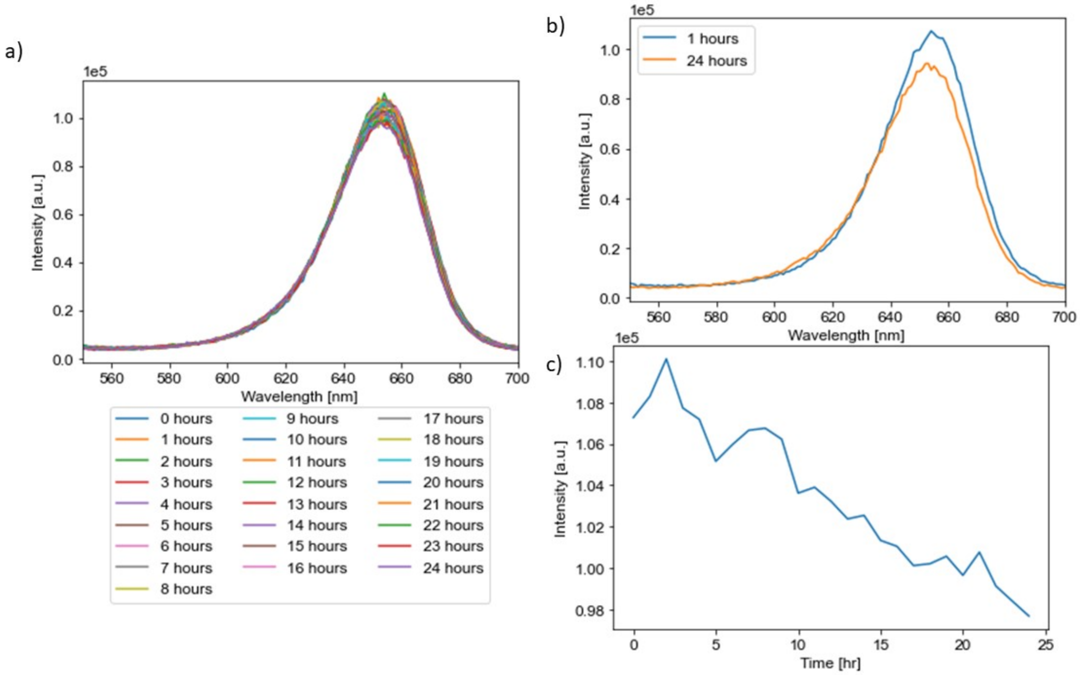

Figure S12: Fort Orange CdSe in PS exposed to DNT. 92.59% quenching. a) fluorescence signals every hour b) 0-hour and 24-hour fluorescence signal c) kinetics of fluorescence over time

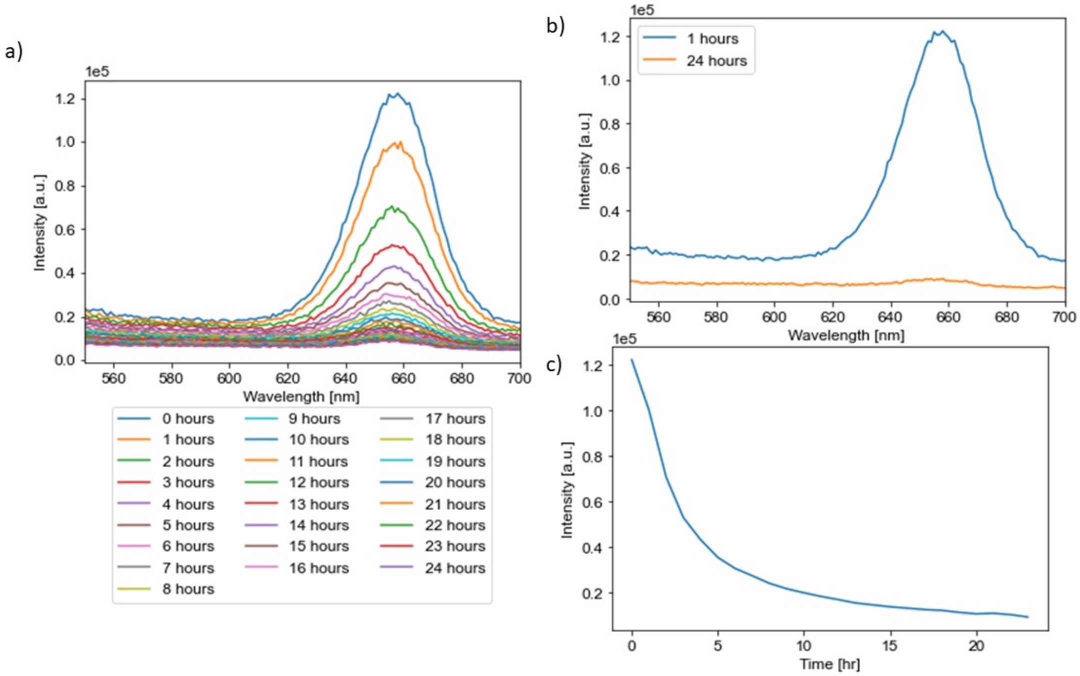

Figure S13: Fort Orange CdSe in PS exposed to TNT. 30.33% quenching. a) fluorescence signals every hour b) 0 hour and 24 hour fluorescence signal c) kinetics of fluorescence over time

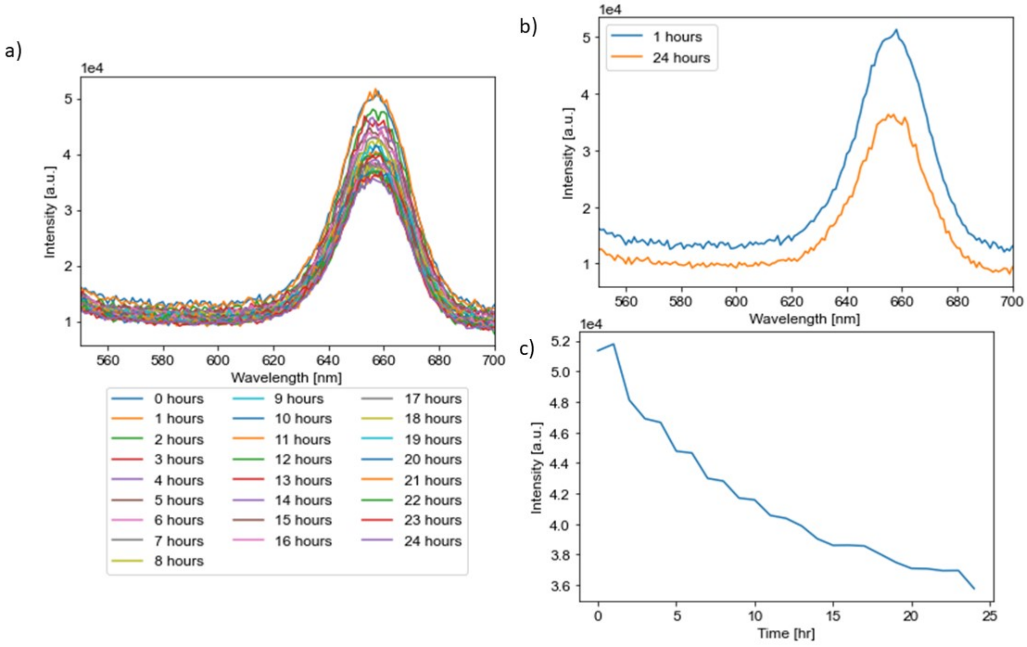

Figure S14: Fort Orange CdSe in PVC exposed to DNT. 75.96% quenching. a) fluorescence signals every hour b) 0 hour and 24 hour fluorescence signal c) kinetics of fluorescence over time

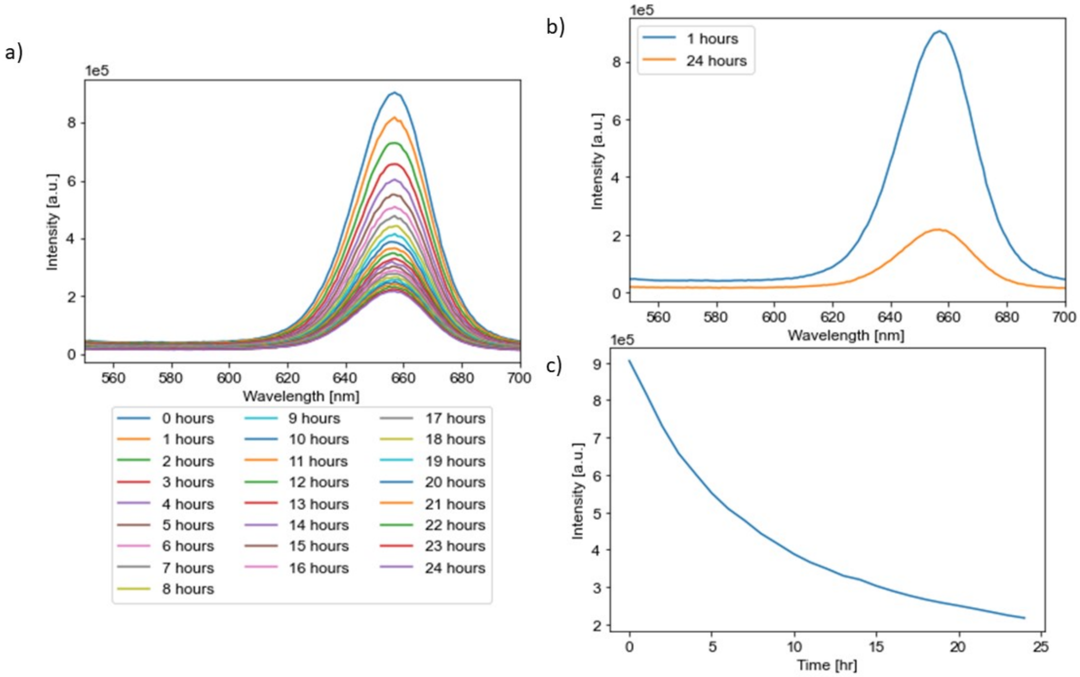

Figure S15: Fort Orange CdSe QDs in PVC exposed to TNT. 20.54% quenching. a) fluorescence signals every hour b) 0-hour and 24-hour fluorescence signal c) kinetics of fluorescence over time

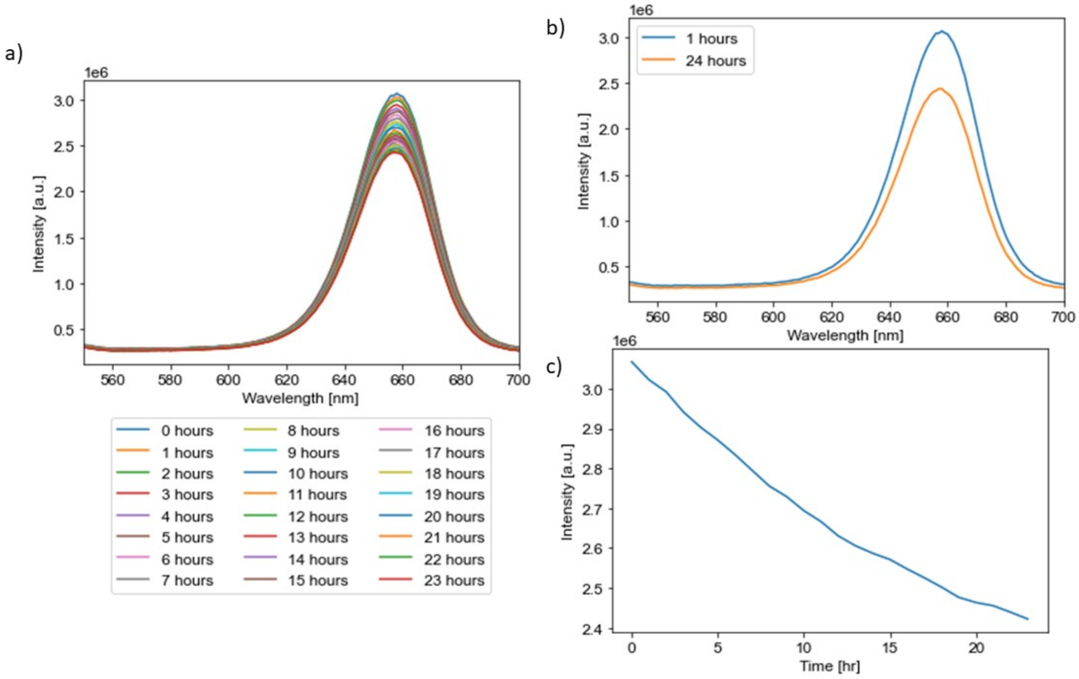

Figure S16: Fluorescence over time for Carbon QDs exposed to TATP, TNT, or DNT

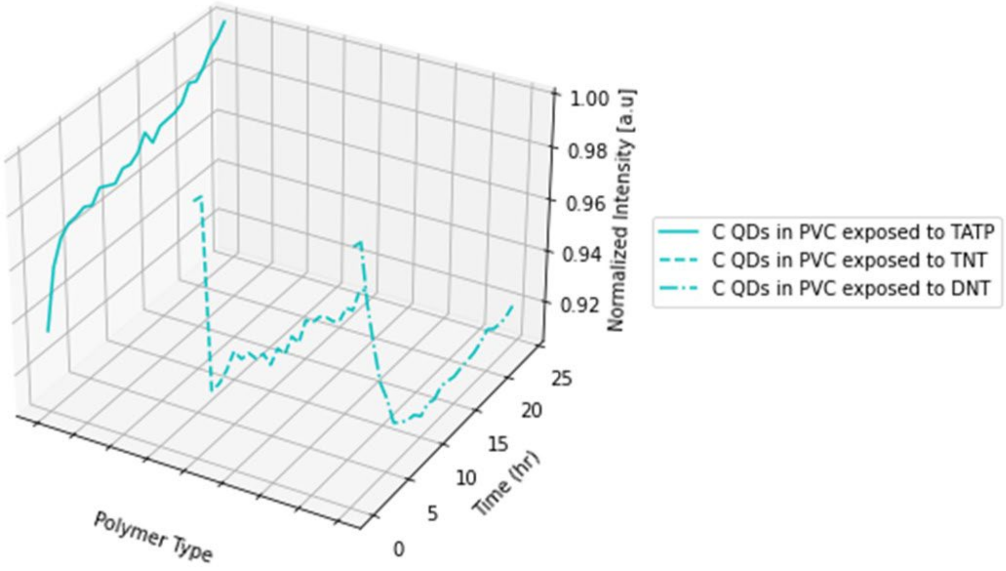

Figure S17: Fluorescence over time for Birch Yellow CdSe exposed to RDX, TATP, TNT, or DNT

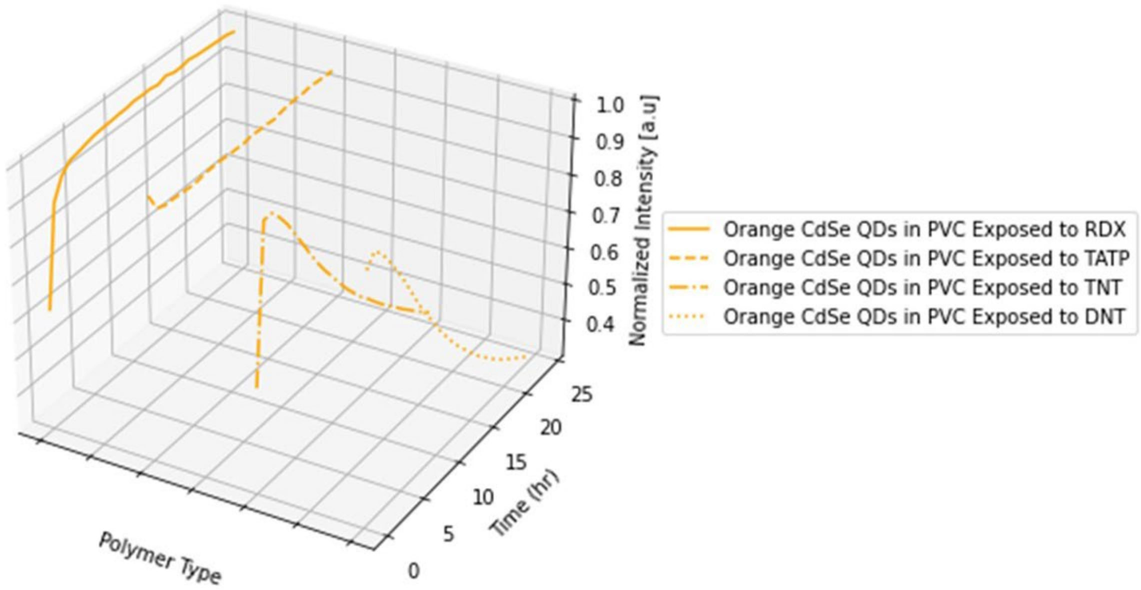

Figure S18: Fluorescence over time for Fort Orange CdSe in PS, PMMA, or PVC exposed to TNT or DNT

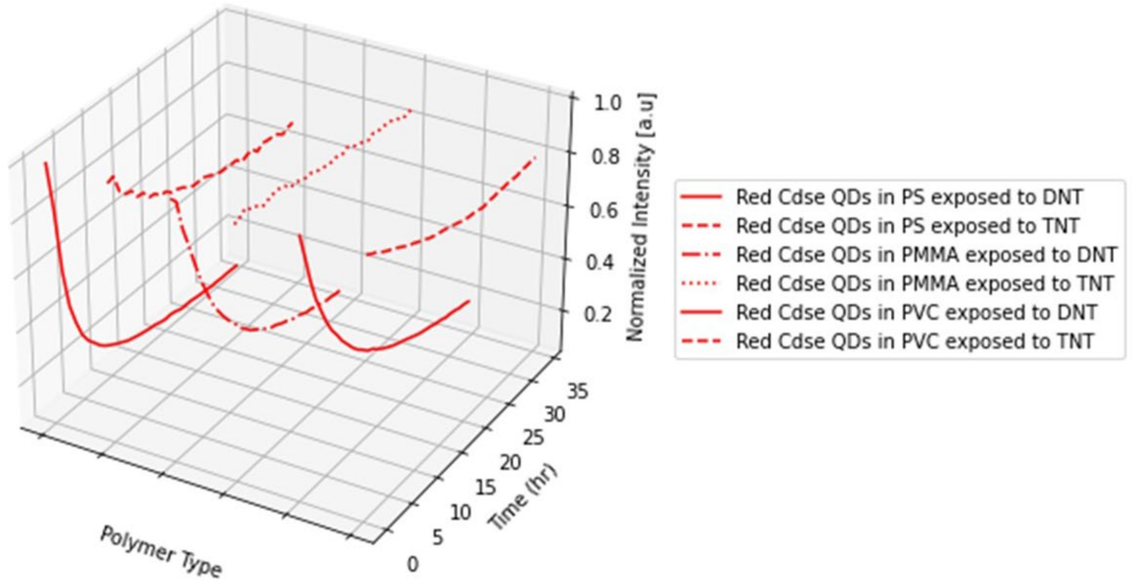

Figure S19: PVC with no QDs exposed to no explosives a) fluorescence signals every hour, b) max and final fluorescence signal

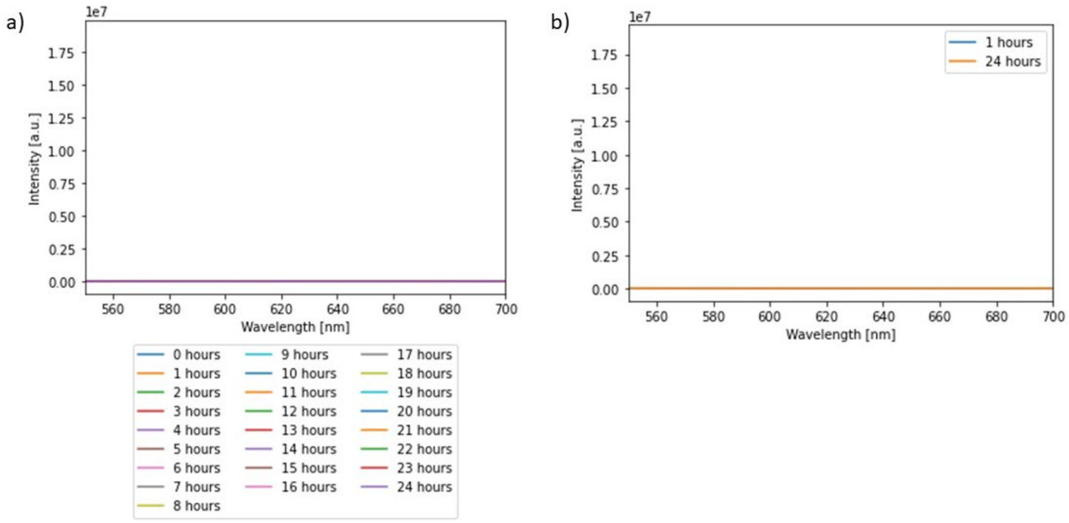

Figure S20: PVC with Birch Yellow CdSe QDs exposed to no explosive a) fluorescence signals every hour b) max and final fluorescence signal

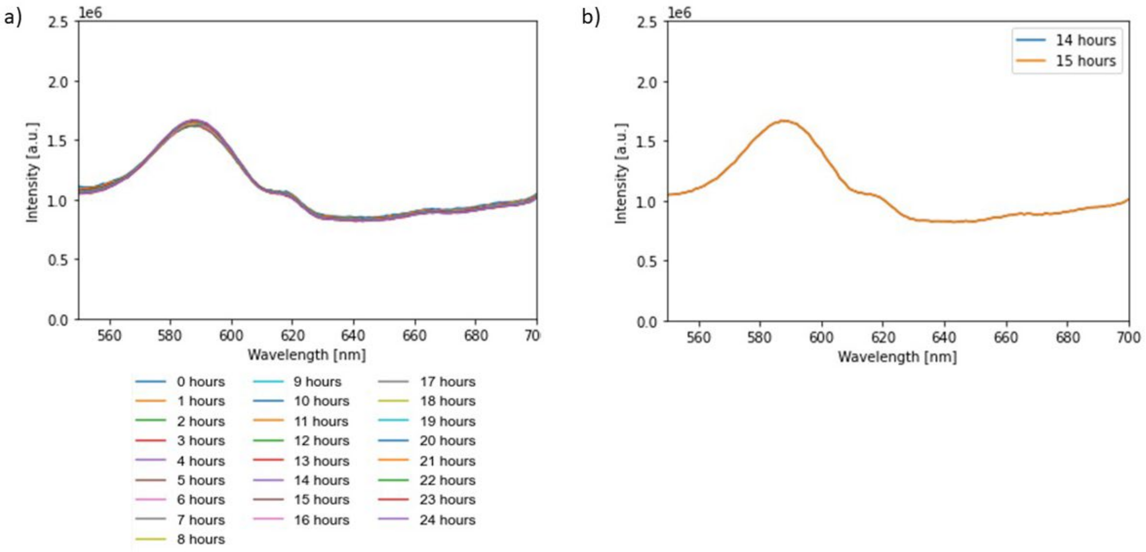

Supplement: Supplementary file 1 — an3c00370_si_001.pdf [file an3c00370_si_001.pdf]
